# Appreciating Factors Beyond the Physical in Talent Identification and Development: Insights From the FC Barcelona Sporting Model

**DOI:** 10.3389/fspor.2020.00091

**Published:** 2020-07-31

**Authors:** Greg Doncaster, Daniel Medina, Franchek Drobnic, Antonio José Gómez-Díaz, Viswanath Unnithan

**Affiliations:** ^1^Edge Hill University, Lancashire, United Kingdom; ^2^Monumental Sports, Washington, DC, United States; ^3^Shanghai Greenland Shenhua Football Club, Shanghai, China; ^4^Football Club Barcelona, Barcelona, Spain; ^5^Division of Sport and Exercise, University of the West of Scotland, Lanarkshire, United Kingdom

**Keywords:** talent identification, talent development, team sports, technical, selection bias

## Abstract

FC Barcelona is a multi-sport organization that adopts a talent identification approach that emphasizes the technical, psychological, and perceptual–cognitive attributes. It is unclear within this type of sporting selection model whether the relative age effect (RAE) exists. Consequently, the aim of the study was to evaluate the RAE across multiple sports and age groups at FC Barcelona. The birthdates of all players (*n* = 6,542) affiliated to each sport [male basketball (*n* = 1,013), male (*n* = 3,012) and female (*n* = 449) soccer, male futsal (*n* = 761), male handball (*n* = 999), and male indoor roller hockey (*n* = 308)] across all age groups from U10 to Senior were recorded. These were then categorized into quartiles from the start of the selection year (Q1 = Jan–Mar; Q2 = Apr–Jun; Q3 = Jul–Sep; Q4 = Oct–Dec) and analyzed for (a) each sport; (b) each age group, irrespective of the sport; and (c) each age group within each sport, using Chi-squared statistics and odds ratios (ORs) with 95% confidence intervals (CIs). Birthdates across the entire club revealed a RAE (Q1 = 46.1%, Q2 = 27.1%, Q3 = 17.1%, and Q4 = 9.7%, χ^2^ = 29.8, *P* < 0.01), with OR in Q1, Q2, and Q3 representing a 4.76 (95% CIs: 1.96–11.57), 2.80 (1.12–7.03), and 1.77 (0.67–4.63) increased likelihood for selection when compared to Q4, respectively. Despite FC Barcelona's approach to talent identification and development, the RAE was still present within youth age groups (<18 years old). The current findings provide support that the RAE is more prevalent within regionally popular sports and reduces with increasing age; however, given the talent identification processes within FC Barcelona's sporting model, additional factors beyond the physical attributes, such as enhanced psychological and perceptual–cognitive attributes, in those born earlier in the selection year might further influence the RAE. Consequently, current results provide indirect evidence to suggest that sociological and psychological determinants might be a greater influence on the presence of RAE in sporting environments that prefer to consider technical and perceptual–cognitive attributes in their talent development programs.

## Introduction

Relative age effects (RAEs) exist when there is a distinct over-representation of players born earlier in the selection year for a given cohort. Researchers suggest that this is due to variations within both the occurrence and rate of growth and maturation within youth sport populations (Malina et al., 2017) when stratified by chronological age groups. Indeed, the RAE was significantly correlated with a team's final rankings in U-17 German first leagues for soccer (Augste and Lames, 2011). Recently, Bezuglov et al. (2019) found the RAE to be highly prevalent within Russian youth soccer and was associated with the level of competitiveness, as the RAE was most pronounced in the top tier of junior Russian soccer academies and national teams. With respect to other sports, Ibañez et al. (2018) found the RAE to be prevalent within elite U-18 basketball, according to position. While the RAE has potential positive implications for success in specific age groups, it has potentially detrimental implications for talent identification and talent development in youth populations. A preference for immediate “success” potentially results in a maturation-selection phenomenon in which there is a preference for selecting early maturers, who display superior physical qualities than their late maturing counterparts (Lovell et al., 2015; Wattie et al., 2015). While the RAE and maturity status should not be viewed as the same construct (Pena-Gonzalez et al., 2018), the unintended consequence is that the less biologically mature individual (those born later in the selection year) is viewed negatively due to their physical disadvantage (Esteva et al., 2006; Lovell et al., 2015; Malina et al., 2017). A potential consequence is that biologically immature, but technically skillful players are overlooked or drop out in the developmental stages of youth development programs and never attain their full potential (Figueiredo et al., 2009; Bezuglov et al., 2019). Physical advantages in strength and power, which are associated with growth and maturation, will have a positive impact on an individual's ability to perform specific movement demands commonly exhibited in team sports (e.g., accelerations, sprints, tackles, and jumps) and are often favored within talent identification and talent development programs (Lovell et al., 2015).

Lovell et al. (2015), however, reported a strong RAE in prepubertal age groups (U9) across numerous English soccer developmental programs, in which the impact of growth and maturation is negligible. This finding is supported by Cobley et al. (2009) meta-analyses, which display a RAE in prepubertal age groups for ice hockey, tennis, and soccer. This suggests that non-physical factors should also be considered when examining the RAE in youth sport. As such, sociological and psychological factors that impact on the prevalence of the RAE should be considered when examining those involved in the selection and developmental processes in high-level youth sport. Indeed, earlier enrolment of relatively older players (players born earlier in the selection year) from a young age is likely to lead to a greater amount of exposure to quality systematic training, formal and expert coaching, and increased access to superior facilities, than their relatively younger counterparts (players born later in the selection year). Moreover, the relationships between youth players and the key stakeholders within the talent identification and development processes should also be considered when exploring RAEs within sport. Research from Larkin and O'Connor (2017) demonstrated that coaches and scouts emphasized the importance of technical attributes in the talent identification and development processes, providing further support that a wider array of factors (beyond the physical) need to be considered to develop an improved understanding of the RAE within youth sport. Consequently, research examining RAEs in sport should benefit from utilizing appropriate theoretical models that enable researchers and practitioners to develop a wider appreciation of the factors that contribute to the prevalence of the RAE, and the interdependent relationships between these factors (Hancock et al., 2013; Wattie et al., 2015). In addition, researchers have often focused on the RAE within a single sport, and while comparisons between published literature can be made, single-study research assessing the presence of the RAE across multiple sports and age groups is limited. Wide-ranging sport clubs (i.e., those that are composed of multiple sports and ages within the same club environment) provide an opportunity to explore the theoretical models proposed by Wattie et al. (2015) and Hancock et al. (2013) within a single, elite sports environment. One such club is FC Barcelona, which includes highly trained athletes, ranging in age from U10 to professional adult status, including (male) basketball, futsal, handball, and indoor roller hockey, as well as male and female soccer.

Previous RAE research might be regarded as atheoretical and limited to observational studies; however, both Wattie et al. (2015) and Hancock et al. (2013) have proposed theoretical models to better explain RAEs in sport. Hancock et al. (2013) propose a social agent model, highlighting the *Matthew* effect, *Pygmalian* effect, and *Galatea* effect as key concepts of the theory that can be used to help explain the influence social agents have on RAEs. For example, Hancock et al. (2013) suggest that initial RAEs (i.e., in younger age groups) are influenced by the Matthew effect, with relatively older players being enrolled (by their parents) in sport at an early age. This results in these children acquiring advanced skills and capabilities (through structured training) in comparison to their relatively younger counterparts and providing coaches with a talent pool that is filled with relatively older athletes (Hancock et al., 2013). Subsequently, the Pygmalian effect highlights the impact that perceived expectations, from others (whether subconsciously or consciously imposed), can have on an individual's outcomes. Finally, once individual expectations have been inferred, the Galatea effect suggests that players will act congruently with these expectations (Hancock et al., 2013). Consequently, Hancock et al. (2013) emphasize the impact social agents might have upon RAEs in sport, arguing that social agents often falsely associate physical maturity with actual performance (skill and physical) differences (Larkin and O'Connor, 2017). Wattie et al. (2015) developed a constraints-based model in which environmental, individual, and task constraints need to be considered to develop an understanding of the existence or non-existence of RAEs. Wattie et al. (2015) constraints-based model suggests that the individual constraints [composed of structural factors (i.e., anthropometric and physical characteristics) and functional factors (i.e., psychological characteristics)], task constraints (i.e., sporting demands), and environmental constraints (i.e., broader social constructs) need to be considered when examining RAEs in sport.

Meta-analyses exploring the RAE in sport have demonstrated that the presence of the RAE appears to be influenced by interactions between developmental stages/age categories, competition/skill level, and sporting demands (i.e., sporting context and type) (Cobley et al., 2009; Smith et al., 2018). Alongside the physical traits that are required for success at the elite level, the FC Barcelona talent identification model emphasizes technical and perceptual–cognitive attributes. This is supported by Gyarmati et al. (2014) evaluation of FC Barcelona's first team's unique style of play in men's soccer, when compared to other top-level European teams. This approach is advocated throughout the youth setup and is a product of synergizing the complex and varied determinants of team sport performance (Mallo, 2020). Therefore, FC Barcelona presents an opportune professional multi-sport model, in which numerous team sports (basketball, soccer, indoor football, handball, and indoor roller hockey) and age groups can be investigated in relation to the theoretical models proposed by Hancock et al. (2013) and Wattie et al. (2015). Consequently, an improved understanding of the prevalence of the RAE within a given sporting context (FC Barcelona), which emphasizes the technical and perceptual–cognitive attributes within their approach to talent identification and development, is likely to have wider implications for the influence of RAE for both talent identification and talent development in other sporting institutions that adopt a similar approach. Specifically, the emphasis on technical and perceptual–cognitive attributes within this context may provide further insights into the extent to which factors beyond the physical, impact upon RAEs in sport.

Therefore, the aim of the present study was to evaluate the RAE across multiple sports at FC Barcelona, which, alongside the required physical traits for elite level success, emphasize the technical and perceptual–cognitive attributes in their talent identification and development processes. This will be done by examining the birthdate distributions within (a) each sport; (b) each age group (U10, U12, U14, U16, U18, and Senior Squads), irrespective of the sport; and (c) each age group within each sport. It was hypothesized that based on FC Barcelona's talent identification model, the RAE would be less prevalent across all age groups and sports than has been previously reported within the RAE literature. However, it was hypothesized that the RAE would remain more prevalent within the younger age groups, relative to the senior squads. Finally, across sports and irrespective of FC Barcelona's talent identification model, it was hypothesized that the RAE would be more prevalent in basketball than other sports, due to the specific sporting (physical) demands, irrespective of the unified club philosophy.

## Materials and Methods

Ethical approval was gained from the Ethics Committee for Clinical Research of the Catalan Sports Council (17/2018 /CEICEGC). The birthdates of all players (*n* = 6,542) affiliated to each sport [male (*n* = 3,012) and female (*n* = 449) soccer, male basketball (*n* = 1,013), male futsal (*n* = 761), male handball (*n* = 999), and male indoor roller hockey (*n* = 308)] within FC Barcelona and across all age groups [U10 (*n* = 588), U12 (*n* = 755), U14 (*n* = 1,488), U16 (*n* = 1,578), U18 (*n* = 944), and Senior (*n* = 1,189)] were recorded. Data from female athletes were only available for soccer. At the senior level, the men's soccer team competes domestically in La Liga and internationally in the UEFA Champions League. The women's soccer team competes domestically in the Primera Division and internationally in the UEFA Women's Champions League. The basketball team competes domestically in Liga ACB and internationally in the EuroLeague. The futsal team competes domestically in the Primera Division and internationally in the UEFA Futsal Cup. The handball team competes domestically in the Liga ASOBAL and internationally in the European Champions Cup. Finally, the indoor roller hockey competes domestically in the OK Liga and internationally in the European League. Finally, all senior teams within each of these sports are recognized as professional teams.

Individuals' birthdates were then categorized into relative age quartiles (Q) from the start of the selection year (Q1 = Jan–Mar; Q2 = Apr–Jun; Q3 = Jul–Sep; Q4 = Oct–Dec). Birthdate distributions across quartiles were then analyzed for (a) each sport; (b) each age group, irrespective of the sport; and (c) each age group within each sport, using Chi squared (χ^2^) statistics and by calculating odds ratios (ORs) and 95% confidence intervals (CIs) for the quartile distributions. The Chi-squared statistic assessed differences between the observed and expected birthdate distributions, with expected birthdate distributions being calculated using available data from birth statistics for Spain, from 2015 and 2016 (Statista, 2018). In accordance with the study by Lovell et al. (2015), reference values were obtained from the general population to provide an indication of the birthdate distribution that participate within grassroots-level sport (i.e., the talent pool from which these players are selected). This resulted in reference values of 24.4, 24.3, 26.1, and 25.1% for Q1, Q2, Q3, and Q4, respectively. Chi-squared statistics, however, do not reveal the magnitude and direction of the existing relationship and therefore ORs were also calculated to examine the bias of birthdate distributions within sub-groups (Q1, Q2, Q3, and Q4). The OR compared the birthdate distribution of a particular quartile (Q1, Q2, or Q3) with the reference group, which consisted of the relatively youngest players (Q4). A higher OR indicates an increased representation of players who were born in that particular quartile compared to the reference quartile Q4 and were considered significant when the CI range did not include a value ≤ 1.00. Finally, where appropriate, the alpha level was set at *P* < 0.05.

## Results

The frequency and percentage distributions of players' birth quartiles within each sport are presented in Table 1. The Chi-squared showed significant deviations across quartiles for all sports, with basketball and male soccer displaying substantially larger χ^2^ values (44.7 and 53.6, respectively) than other sports. All sports, excluding female soccer, displayed the highest percentage distribution of birthdates within Q1, with a progressive decline to Q4. Female soccer, however, displayed a larger percentage distribution of birthdates within Q2 (32.5%), when compared to Q1 (30.1%). OR revealed that basketball and male soccer had higher and comparable RAE, with female soccer, futsal, handball, and roller hockey demonstrating weaker, though significant, effects.

**Table 1:** Birth quartiles by sport across all age groups.

		**Birthdate distribution # (%)**				**Odds ratios (95% CI)**			**Chi-squared**
**Sport**	***n***	**Q1**	**Q2**	**Q3**	**Q4**	**Q1 vs. Q4**	**Q2 vs. Q4**	**Q3 vs. Q4**	**χ^**2**^**
Basketball	1,013	506 (50.0)	279 (27.5)	157 (15.5)	71 (7.0)	7.1 (2.7–18.7)	3.9 (1.5–10.7)	2.2 (0.8–6.3)	44.7^*^
Soccer (Male)	3,012	1599 (53.1)	798 (26.5)	422 (14.0)	193 (6.4)	8.3 (3.1–22.3)	4.1 (1.5–11.5)	2.2 (0.7–6.5)	53.6^*^
Soccer (Female)	449	138 (30.7)	146 (32.5)	90 (20.0)	75 (16.7)	1.8 (0.8–4.1)	1.9 (0.9–4.4)	1.2 (0.5–2.8)	8.7^*^
Futsal	761	297 (39.0)	189 (24.8)	170 (22.3)	105 (13.8)	2.8 (1.2–6.5)	1.8 (0.8–4.3)	1.6 (0.7–3.9)	14.5^*^
Handball	999	363 (36.3)	273 (27.3)	212 (21.2)	151 (15.1)	2.4 (1.1–5.4)	1.8 (0.8–4.2)	1.4 (0.6–3.3)	11.1^*^
Roller hockey	308	106 (34.4)	86 (27.9)	74 (24.0)	42 (13.6)	2.5 (1.1–5.8)	2.0 (0.9–4.8)	1.8 (0.7–4.2)	10.1^*^

* *Significant effect at an alpha level of P < 0.05. Q1 = Jan–Mar, Q2 = Apr–Jun, Q3 = Jul–Aug, and Q4 = Sep–Oct*.

The frequency and percentage distributions of players' birth quartiles within each age group are presented in Table 2. The χ^2^ showed significant deviations across quartiles for all youth age groups (U10, U12, U14, U16, and U18). Within the senior squads, however, there was no significant difference between the birthdate distribution of senior players and the expected birthdate distributions (χ^2^ = 4.2, *P* = 0.24). In all age groups, Q1 birthdates were most overrepresented, with a progressive decline to Q4—though the inter-quartile differences dissipated with increasing age. Analysis of the RAE within each age group, using OR analysis, revealed that the RAE was most prevalent within the U10 age group, with U12 to U18 age groups still displaying a RAE, although there was shown to be a reduction in OR values between Q1, Q2, Q3, and the reference value of Q4, with increasing age.

**Table 2:** Birth quartiles by age groups across all sports.

		**Birthdate distribution # (%)**				**Odds ratios (95% CI)**			**Chi-squared**
**Age group**	***n***	**Q1**	**Q2**	**Q3**	**Q4**	**Q1 vs. Q4**	**Q2 vs. Q4**	**Q3 vs. Q4**	**χ^**2**^**
U10	588	327 (55.6)	177 (30.1)	66 (11.2)	18 (3.1)	18.2 (5.1–65.2)	9.8 (2.7–36.1)	3.7 (0.9–14.6)	69.2^*^
U12	755	376 (49.8)	220 (29.1)	101 (13.4)	58 (7.7)	6.5 (2.5–16.6)	3.8 (1.4–10.0)	1.7 (0.6–5.0)	45.7^*^
U14	1,488	737 (49.5)	401 (26.9)	228 (15.3)	122 (8.2)	6.0 (2.4–15.2)	3.3 (1.3–8.6)	1.9 (0.7–5.0)	42.0^*^
U16	1,578	756 (47.9)	430 (27.2)	256 (16.2)	136 (8.6)	5.6 (2.2–13.9)	3.2 (1.2–8.1)	1.9 (0.7–5.1)	38.7^*^
U18	944	437 (46.3)	243 (25.7)	173 (18.3)	91 (9.6)	4.8 (2.0–11.7)	2.7 (1.1–6.7)	1.9 (0.7–5.0)	31.7^*^
Senior	1,189	376 (31.6)	300 (25.2)	301 (25.3)	212 (17.9)	1.8 (0.8–3.9)	1.4 (0.6–3.2)	1.4 (0.6–3.3)	4.2

* *Significant effect at an alpha level of P < 0.05. Q1 = Jan–Mar, Q2 = Apr–Jun, Q3 = Jul–Aug, and Q4 = Sep–Oct*.

The frequency and percentage distributions of players' birth quartiles within each age group and each sport are presented in Table 3. According to the χ^2^ statistic, the RAE was present within all youth age groups (U18 and below) and across all sports excluding the roller hockey U12 age group, in which there was a substantially smaller number of players. In senior squads, the χ^2^ statistic was shown to be significant within basketball, male soccer, and roller hockey; however, the value of the χ^2^ statistic in all senior squads was lower than the χ^2^ values provided in the younger age groups, for the respective sports. OR analysis supported these findings, demonstrating that there is a consistent bias toward individuals born early in the selection year, particularly within those born within Q1. The largest OR values were found in the younger age groups within basketball and male soccer, in which there were a higher *number of players in each sport*. The OR values within female soccer, futsal, handball, and roller hockey also support the presence of the RAE; however, the extent to which there is a bias (e.g., the size of the OR) for those born earlier within the selection year was lower than those found in basketball and soccer.

**Table 3:** Birth quartiles by sport and age groups.

**All sport**	**Age group**		**Birthdate distribution # (%)**				**Odds ratios (95% CI)**			**Chi-squared**
		***n***	**Q1**	**Q2**	**Q3**	**Q4**	**Q1 vs. Q4**	**Q2 vs. Q4**	**Q3 vs. Q4**	**χ^**2**^**
Basketball	U10	*No Squad*								
	U12	*No Squad*								
	U14	333	182 (54.7)	95 (28.5)	42 (12.6)	14 (4.2)	13.0 (4.2–40.1)	6.8 (2.1–21.7)	3.0 (0.9–10.3)	62.8^*^
	U16	303	172 (56.8)	81 (26.7)	38 (12.5)	12 (4.0)	14.3 (4.5–45.7)	6.8 (2.1–22.2)	3.2 (0.9–11.1)	68.2^*^
	U18	140	72 (51.4)	36 (25.7)	25 (17.9)	7 (5.0)	10.3 (3.5–30.1)	5.1 (1.7–15.6)	3.6 (1.2–11.1)	48.7^*^
	Senior	237	80 (33.8)	67 (28.3)	52 (21.9)	38 (16.0)	2.1 (0.9–4.8)	1.8 (0.8–4.0)	1.4 (0.6–3.2)	8.3^*^
Soccer (Male)	U10	588	327 (55.6)	177 (30.1)	66 (11.1)	18 (3.1)	18.2 (5.1–65.2)	9.8 (2.7–36.1)	3.7 (0.9–14.5)	69.3^*^
	U12	573	308 (53.8)	167 (29.1)	64 (11.2)	34 (5.9)	9.1 (3.3–24.5)	4.9 (1.7–13.9)	1.9 (0.6–5.9)	59.6^*^
	U14	561	307 (54.7)	147 (26.2)	66 (11.8)	41 (7.3)	7.5 (2.9–19.4)	3.6 (1.3–9.7)	1.6 (0.6–4.7)	58.3^*^
	U16	535	301 (56.3)	126 (23.6)	77 (14.4)	31 (5.8)	9.7 (3.5–26.9)	4.1 (1.4–11.8)	2.5 (0.8–7.6)	61.9^*^
	U18	436	228 (52.3)	101 (23.2)	77 (17.7)	30 (6.9)	7.6 (2.9–20.0)	3.4 (1.2–9.3)	2.6 (0.9–7.3)	47.9^*^
	Senior	319	128 (40.1)	80 (25.1)	72 (22.6)	39 (12.2)	3.3 (1.4–7.7)	2.1 (0.9–4.9)	1.9 (0.8–4.5)	17.3^*^
Soccer (Female)	U10	*No Squad*								
	U12	*No Squad*								
	U14	115	46 (40.0)	37 (32.3)	18 (15.7)	14 (12.2)	3.3 (1.4–7.7)	2.6 (1.1–6.3)	1.3 (0.5–3.3)	23.5^*^
	U16	133	41 (30.8)	47 (35.3)	26 (19.5)	19 (14.3)	2.2 (0.9–5.0)	2.5 (1.1–5.7)	1.4 (0.6–3.3)	13.0^*^
	U18	*No Squad*								
	Senior	201	51 (25.4)	62 (30.8)	46 (22.9)	42 (20.9)	1.2 (0.5–2.7)	1.5 (0.7–3.2)	1.1 (0.5–2.5)	2.9
Futsal	U10	*No Squad*								
	U12	155	60 (38.7)	45 (29.0)	33 (21.3)	17 (11.0)	3.5 (1.5–8.4)	2.7 (1.1–6.4)	1.9 (0.8–4.9)	18.2^*^
	U14	161	74 (46.0)	40 (24.8)	33 (20.5)	14 (8.7)	5.3 (2.1–13.2)	2.9 (1.1–7.4)	2.4 (0.9–6.2)	31.1^*^
	U16	140	63 (45.0)	44 (31.4)	23 (16.4)	10 (7.1)	6.3 (2.4–16.5)	4.4 (1.7–11.8)	2.3 (0.8–6.5)	36.1^*^
	U18	143	56 (39.2)	36 (25.2)	28 (19.6)	23 (16.1)	2.4 (1.1–5.4)	1.6 (0.7–3.6)	1.2 (0.5–2.9)	13.9^*^
	Senior	162	44 (27.2)	24 (14.8)	53 (32.7)	41 (25.3)	1.1 (0.5–2.3)	0.6 (0.3–1.4)	1.3 (0.6–2.8)	5.7
Handball	U10	*No Squad*								
	U12	*No Squad*								
	U14	238	94 (39.5)	60 (25.2)	52 (21.8)	32 (13.4)	2.9 (1.3–6.7)	1.9 (0.8–4.5)	1.6 (0.7–3.9)	15.8^*^
	U16	398	151 (37.9)	112 (28.1)	80 (20.1)	55 (13.8)	2.8 (1.2–6.3)	2.0 (0.9–4.8)	1.5 (0.6–3.5)	14.6^*^
	U18	150	59 (39.3)	47 (31.3)	23 (15.3)	21 (14.0)	2.8 (1.2–6.4)	2.2 (1.0–5.2)	1.1 (0.4–2.7)	20.5^*^
	Senior	213	59 (27.7)	54 (25.4)	57 (26.8)	43 (20.2)	1.4 (0.6–3.1)	1.3 (0.6–2.8)	1.3 (0.6–3.0)	1.5
Roller hockey	U10	*No Squad*								
	U12	27	8 (29.6)	8 (29.6)	4 (14.8)	7 (25.9)	1.1 (0.5–2.5)	1.1 (0.5–2.5)	0.6 (0.3–1.3)	7.2
	U14	80	34 (42.5)	22 (27.5)	17 (21.3)	7 (8.8)	4.9 (2.0–12.1)	3.1 (1.2–8.1)	2.4 (0.9–6.4)	25.4^*^
	U16	69	28 (40.6)	20 (29.0)	12 (17.4)	9 (13.0)	3.1 (1.4–7.2)	2.2 (0.9–5.2)	1.3 (0.5–3.3)	20.5^*^
	U18	75	22 (29.3)	23 (30.7)	20 (26.7)	10 (13.3)	2.2 (0.9–5.2)	2.3 (1.0–5.4)	2.0 (0.9–4.7)	8.3^*^
	Senior	57	14 (24.6)	13 (22.8)	21 (36.8)	9 (15.8)	1.6 (0.7–3.6)	1.4 (0.6–3.4)	2.3 (1.0–5.2)	8.0^*^

* *Significant effect at an alpha level of P < 0.05*.

## Discussion

The major findings from this novel investigation were that (a) the RAE is present within a range of sports, but that it is most prevalent in sports that may be regarded as regionally popular (e.g., basketball and male soccer), and (b) the RAE is apparent within all youth age groups (U10–U18s), becoming less prevalent with increasing age, and then negligible within senior squads. Consequently, both sport and age appear to be key factors with regard to the presence and extent of the RAE and should be considered when undertaking research within RAEs in sport and when applying any theoretical models. This pattern has emerged irrespective of the underlying club philosophy, which emphasizes the technical and perceptual–cognitive characteristics of the player. Indeed, current findings may suggest that there is a bias toward individuals who are born early in the selection year, despite club talent identification (and development) criteria that favor the more “technically” dominant athlete. It is possible that physical attributes might still be a determinant within the talent identification and development model, despite the overarching technique-based recruitment model of FC Barcelona. Equally, it could be postulated that social agents as well as individual, environmental, and task constraints, proposed in the theoretical models of Hancock et al. (2013) and Wattie et al. (2015), falsely exacerbate the prevalence of the RAE in which physical attributes are a contributing factor.

Within the current study, the RAE was greater within basketball and male soccer, as evidenced by the χ^2^ statistic and the OR analysis. Furthermore, while the extent of the RAE decreased with increasing age, the χ^2^ statistics still revealed a significant difference between the birthdate distribution of senior players and the expected birthdate distributions (across birth quartiles) for both basketball (χ^2^ = 8.3, *P* = 0.03) and male soccer (χ^2^ = 17.3, *P* = 0.0006) players. OR analysis within these sports suggests that the RAE was more prevalent within the younger age groups; however, the percentage distribution of birthdates across quartiles should be acknowledged, as a smaller percentage within Q4 will inflate the OR values for Q1, Q2, and Q3. All youth age groups in basketball and male soccer displayed a percentage distribution of birthdates within Q1 that was >50%, suggesting a consistent bias toward selecting those individuals born earlier in the selection year (i.e., chronologically older individuals). Specifically, the more prevalent RAE in basketball could be linked to the fact that position specificity is an issue even at a young age, with individuals of high stature being favored. Sallett et al. (2005) demonstrated that all positions within basketball, other than the point guard where technical (ball handling) skills are key, emphasize stature as a pre-requisite and therefore taller players tend be selected, thus favoring those born earlier in the selection year, leading to an anthropometric manifestation of the RAE (Sallett et al., 2005). Likewise, selection processes in soccer (talent identification) often favor those with superior levels of physicality (Lovell et al., 2015), as well as those of increased stature within certain positions (e.g., goalkeeper and central defender). Again, this results in a bias toward those born earlier in the selection year (Q1 and Q2), as such individuals are likely, but not necessarily (Pena-Gonzalez et al., 2018), to be of increased growth and advanced maturity, in comparison to individuals born later in the selection year (Q3 and Q4). In contrast, recent research from Ibañez et al. (2018) found the RAE among U18 basketball players to be most prevalent within the guard position and least prevalent within centers. This suggests that physical prowess and anthropometric advantages might not be as influential as initially thought, as guards need high levels of technical, tactical, and perceptual–cognitive abilities (Ibañez et al., 2018). Consequently, an increased prevalence of the RAE within this position suggests that relatively older players might enhance their sport-specific performance skills faster than their relatively younger peers. This might be a result of an increased exposure to sport-specific motor experiences, increased exposure to quality coaches and facilities as well as regular involvement in higher competition levels from a younger age, which are a consequence of a selection bias of those born earlier in the year within younger age groups (Ibañez et al., 2018). In support of this, Figueiredo et al. (2019) found limited differences between players, across birth quartiles, for functional capacities, soccer skills, goal orientation, and coach evaluation of potential; yet, coaches tended to rate players born in Q1 as higher in potential. This aligns with the context of the current study, as the FC Barcelona club philosophy emphasizes technical, psychological, and perceptual–cognitive characteristics rather than prioritizing physical precocity; however, the RAE is still prevalent within this context.

In combination with previous research, current results might be explained in accordance with the theoretical models proposed by Hancock et al. (2013) and Wattie et al. (2015), which propose theoretical frameworks that highlight key factors that contribute to the false association between physical maturity and actual performance. Within the current study, findings from female soccer, futsal, handball, and roller hockey displayed a lower RAE in comparison to basketball and male soccer and were more inconsistent with regard to the presence of the RAE across age groups. Both futsal and handball are played on a smaller playing surface (Barbero-Alvarez et al., 2008; Povoas et al., 2012), which might reduce the emphasis on physicality in comparison to other sports. This could diminish the individual and environmental constraints, resulting in a reduced prevalence of the RAE, when compared to basketball and soccer. Conversely, the importance of technical proficiency within soccer (Helsen et al., 2005; Larkin and O'Connor, 2017) and basketball (Ibañez et al., 2018) should not be underestimated, particularly given the emphasis on “technique” within the underlying club philosophy in the current study. Consequently, the individual and environmental constraints within these sports might exacerbate the RAE (Wattie et al., 2015).

Theoretical frameworks provided by Hancock et al. (2013) and Wattie et al. (2015) might be adapted depending on the context in which the RAE is being examined. The less prevalent RAEs stated above may be a consequence of a reduction in the extent to which social agents (Hancock et al., 2013) or “constraints” (Wattie et al., 2015) influence the prevalence of the RAE. For example, in sports of reduced popularity, the Matthew effect is likely to be less evident, as these are not “sports” that appear to be encountered at an early age (as evidenced in Table 3). Therefore, the reduced popularity of a sport may diminish the extent to which social agents influence the talent identification and developmental processes (from an early age), resulting in a smaller and more equitable talent pool to draw from Cobley et al. (2009) and Hancock et al. (2013), and a subsequent reduction in the RAE. In addition, sports regarded as regionally popular are likely to lead to increased levels of competitiveness from an early age. As such, increased levels of competitiveness have been shown to exacerbate the RAE (Bezuglov et al., 2019) and this might be due to the manner in which the social agents involved in the talent identification and development processes interpret and implement a club's philosophy. Therefore, within the current context, despite the unified approach to talent identification and development, the actual implementation of this approach might differ from one sport to another, as a result of the sport-specific contextual factors (e.g., competitiveness). Consequently, theoretical models and future research seeking to better explain the RAE should consider whether the influence of the proposed contributing factors (i.e., social agents or theories, growth and maturation, and physical prowess) alters depending on the contextual factors (i.e., age, sport, gender, and playing level). Indeed, the current results are supported by Cobley et al. (2009) meta-analysis, which also found the highest Q1:Q4 OR in basketball and soccer, when compared to ice hockey, baseball, volleyball, and American football. However, given the large number of studies compared within the meta-analysis by Cobley et al. (2009) and the differing sporting contexts and cultures, there is an inability to gain an understanding of the popularity or profile of each sport, and the surrounding contextual factors, within each of the respective studies. As such, the current paper supports and extends on the existing literature and provides indirect evidence that supports the use and application of the theoretical models proposed by Hancock et al. (2013) and Wattie et al. (2015).

Present results demonstrated a persistent, but not universal, bias toward selecting individuals born early in the selection year, particularly within the younger age groups and more high-profile sports. Current results also demonstrate that the presence of the RAE diminished with increasing age, particularly within senior squads, a finding that is supported by previous research (Helsen et al., 2005; Esteva et al., 2006; Lovell et al., 2015). Indeed, similar to the present findings, Lovell et al. (2015) reported smaller OR between Q1 and Q4 in U17 and U18 soccer players, in comparison to younger age groups (U9–U16). Incidentally, while the composition of the theoretical models proposed by Hancock et al. (2013) and Wattie et al. (2015) remains stable with increasing age, the variance to which the specific social agents (and theories) or constraints impacts upon one individual to another is likely to reduce with increasing age. For example, within younger age groups, particularly in high-profile sports, the Pygmalian and Galatea effects might be particularly prevalent at both ends of the spectrum (positive and negative), due to the likely variation in a range of abilities resulting in the presence of low and high expectations. However, as players age, the impact of the Pygmalian and Galatea effects is likely to be reduced as the variation in ability is decreased and the majority of coaches and players will have high expectations, ultimately leading to professional adult status where all involved should be able to perform at a high level and have high expectations. However, the smaller disparities in growth and maturation, comparable levels in physical performance (Lovell et al., 2015), and the greater levels of exposure to training with increasing age should also be considered in relation to the reduced prevalence of the RAE.

As the RAE is also witnessed in education (Jeronimus et al., 2015), there are implications regarding learning capabilities in sport (Pena-Gonzalez et al., 2018). The philosophy of FC Barcelona (i.e., focus on technical and perceptual–cognitive attributes) means that key performance attributes are related to learning capabilities (e.g., decision-making and game intelligence); yet, these are understudied variables in RAE research. This provides further support for adopting a theoretical based approach to examining the RAE, and a wider appreciation of the extent to which the contextual factors and social agents impact upon the prevalence of the RAE. Furthermore, it proposes potential implications with regard to the learning capabilities and more specifically information processing, which in turn would have implications for the prevalence of the RAE in relation to talent development and identification programs within sporting contexts (Pena-Gonzalez et al., 2018). Key attributes that influence selection within the current context include decision-making, technical proficiency, and game intelligence (e.g., cognition and spatial awareness); however, there is a paucity of research that has investigated such variables in association with the prevalence of the RAE. Huertas et al. (2019) however, examined the “cognitive function” of youth soccer players within two elite academies in relation to birth quartile. Despite the presence of the RAE, measures assessing players' cognitive function were comparable across birth quartiles (Huertas et al., 2019). Similar to existing RAE research in elite settings though, players' abilities (e.g., physical or cognitive measures) were shown to be comparable across birth quartiles, yet the RAE remained prevalent. As such, future research should seek to examine the pool of players from which the academy players are selected, as it appears that specific standards and expectations (imposed by social agents) are needed to reach the Academy level, yet the extent to which players from different birth quartiles meet these standards is disproportionate. Moreover, research examining the RAE in association with the talent identification and development processes has tended to adopt a reductionist approach, in which distinct characteristics associated with superior performance are assessed and analyzed in isolation (Unnithan et al., 2012). Consequently, research examining the RAE within sport should seek to assess beyond the physical and develop a wider appreciation of the RAE, ideally at the point at which grassroots-level participation leads into the Academy level (i.e., highly trained youth athletes). In doing so, future RAE research should also seek to develop an evaluative model, which begins to examine the synergy between key characteristics, associated with successful sporting performance.

The absence of physical performance data and perceptual–cognitive abilities is a limitation of the current study. In this regard, however, the purpose of the current study was to examine the extent of the RAE within a range of sports and across multiple age groups, in an internationally recognized multi-sport club that has a unique, overarching philosophy that emphasizes the technical, psychological, and perceptual–cognitive markers for talent identification, alongside the physical traits that are required for success at the elite level. In doing so, this research has demonstrated that, irrespective of the technical focus, the RAE is still present and is more prevalent within the younger age groups and in sports that are regarded as regionally popular (Hancock, 2020). Further investigations, however, are required to explore the relationships and differences in physical, perceptual–cognitive, and psychological characteristics, in association with the RAE. The implications of such research could provide insight for both talent development and talent identification processes, particularly within younger populations. Indeed, an improved understanding of the prevalence of the RAE within a given sporting context (FC Barcelona), which emphasizes the technical and perceptual–cognitive approach to talent identification, is likely to have wider implications for the meaning of the RAE for both talent identification and talent development in other sporting institutions, particularly those that adopt a similar approach. The difficulty in conducting such research, however, is developing a study design and data collection procedures that can obtain a holistic overview. Nevertheless, this identifies avenues for further research and highlights the need for an improved understanding toward the factors (and underpinning theories) influencing the RAE and, in particular aspects, beyond the physical. Consequently, research examining RAEs in sport should seek to explore the psychological (e.g., self-confidence, concentration, attention, and anxiety), perceptual–cognitive (e.g., decision-making, motor/technical skills, and game intelligence/awareness), and social (e.g., interaction between coaches, parents, and athletes) characteristics using an integrated approach (Figueiredo et al., 2019).

## Conclusion

Results from this study demonstrate a persistent, but not universal, bias toward selecting individuals born early in the selection year, particularly within the younger age groups and in sports that are regarded as “high profile” (basketball and male soccer). Furthermore, the presence of the RAE diminished with increasing age, with smaller OR values evident within the senior squads. As a result, while there are numerous additional factors that require further investigation, the current results suggest that the popularity of sports in FC Barcelona affects the prevalence of the RAE. Indeed, application of the theoretical models proposed by Hancock et al. (2013) and Wattie et al. (2015) provides an improved framework for investigating the RAE within sporting contexts. The present results and context of the current study pose interesting questions regarding the factors that result in the presence of the RAE, within elite level sport. Indeed, due to the overarching club philosophy at FC Barcelona for superior psychological and perceptual–cognitive markers, the extent of the RAE might go beyond the previously researched physical dominance of those born earlier within the selection year. Nevertheless, further research should consider a greater array of the potential factors (i.e., social agents or theories, growth and maturation, physical prowess, and psychological and perceptual–cognitive markers) contributing to the prevalence of the RAE, and whether these alter across varying contexts (i.e., sport, age, playing level, and popularity).

## Data Availability Statement

The datasets generated for this study will not be made publicly available because they were provided by the club in confidentiality.

## Ethics Statement

The studies involving human participants were reviewed and approved by Ethics Committee for Clinical Research of the Catalan Sports Council (17/2018/CEICEGC). Gatekeeper approval for the use of anonymised data was provided by FC Barcelona.

## Author Contributions

DM and FD were involved in collecting the necessary data. GD performed the data analysis and completed the initial draft of the manuscript. VU managed the project and aided in the analysis and development of the manuscript. All authors reviewed the paper prior to submission.

## Conflict of Interest

AG-D is employed by FC Barcelona. FD and DM, who were also working during the development of the study at FC Barcelona, have now moved to Shanghai Greenland FC and Monumental Sports, USA, respectively. The remaining authors declare that the research was conducted in the absence of any commercial or financial relationships that could be construed as a potential conflict of interest.
